# Enlarged Perivascular Spaces (EPVS) and the Risk of Amyotrophic Lateral Sclerosis (ALS): Evidence for Overlapping Genetic Signals in White Matter Without Causal Links

**DOI:** 10.3390/brainsci16020144

**Published:** 2026-01-28

**Authors:** Xin Huang, Kailin Xia, Shan Ye, Qiong Yang, Dongsheng Fan

**Affiliations:** 1 Department of Neurology, Peking University Third Hospital, 49 North Garden Road, Haidian District, Beijing 100191, China; 2311110546@bjmu.edu.cn (X.H.); kllook@pku.edu.cn (K.X.); 1263185463@pku.edu.cn (S.Y.); dsfan2010@aliyun.com (D.F.); 2 Key Laboratory for Neuroscience, National Health Commission/Ministry of Education, Peking University, Beijing 100191, China; 3 Beijing Key Laboratory of Biomarker and Translational Research in Neurodegenerative Diseases, Beijing 100053, China

**Keywords:** amyotrophic lateral sclerosis, perivascular spaces, shared genetics, genetic correlation, mendelian randomization

## Abstract

**Background/Objectives**: Emerging evidence suggests that enlarged perivascular spaces (EPVS), which play a significant role in brain fluid exchange and waste removal, may be involved in the pathogenesis of amyotrophic lateral sclerosis (ALS). In this study, we aimed to explore the shared genetic link and causal effect between EPVS and ALS. **Methods**: This study used publicly available summary data from the largest and most recent genome-wide association studies (GWAS) on EPVS (*n* = 40,095) and ALS (*n* = 138,086) in European populations. EPVS were assessed in the hippocampus (EPVS-HIP), basal ganglia (EPVS-BG), and white matter (EPVS-WM). We used linkage disequilibrium score regression (LDSC) to investigate the genetic correlation. Multi-trait analysis of GWAS (MTAG), Cross-Phenotype Association (CPASSOC) analysis, and genetic colocalization analysis were performed to identify shared risk loci. Bidirectional Mendelian randomization analysis was used to investigate the causal relationship. **Results**: A negative genetic correlation was observed between EPVS-WM and ALS after Bonferroni correction (rg = −0.24, *p* < 0.01). No significant correlations were observed between ALS and EPVS-HIP (rg = −0.03, *p* = 0.79) or EPVS-BG (rg = 0.01, *p* = 0.91). Four significant loci including rs113247976 in *KIF5A* and rs118082508 in *SDR9C7* were identified as potential pleiotropic loci of the relationship. None of these loci demonstrated evidence of genetic colocalization. Furthermore, Mendelian randomization analysis revealed no causative effect in either direction. **Conclusions**: EPVS-WM and ALS may share part of their genetic architecture, but no evidence for a causal relationship was observed. Future research is needed to further refine these relationships.

## 1. Introduction

Amyotrophic lateral sclerosis (ALS) is a progressive disorder characterized by the degeneration of motor neurons in the brain and spinal cord [1]. Most patients with ALS die within 3 to 5 years after disease onset. ALS has a strong genetic basis and its genetic architecture is highly complex [2,3]. Approximately 10% of patients exhibit a family history of this disease. The heritability estimates of ALS range from 40% to 60% based on twin studies and population-based cohorts [4,5,6,7,8]. In recent decades, more than 40 genes have been demonstrated to be associated with the risk of ALS [3]. The latest and largest cross-ancestry genome-wide association study (GWAS), including 29,612 cases and 122,656 controls, identified 15 genetic risk loci for ALS [9]. Furthermore, Zhang et al. and Megat et al. integrated GWAS with functional genomic and proteomic datasets, thereby identifying more candidate genes and key pathways [3,10]. Despite these advances, a considerable part of the genetic architecture of ALS remains unknown.

Perivascular spaces (PVS) are anatomical spaces around small vessels presenting as small, linear hyperintensities on T2-weighted images and hypointensities on T1-weighted images on magnetic resonance imaging (MRI) [11,12]. Current evidence has suggested that as the anatomical structure of the glymphatic system, PVS play a significant role in brain fluid exchange and waste removal [13,14,15]. Enlarged PVS (EPVS) are considered to be a marker of perivascular space dysfunction [11,16,17]. Previous studies have suggested that EPVS may be involved in the pathogenesis of ALS [18,19,20,21,22]. Månberg et al. observed EPVSduring the presymptomatic stage of sporadic ALS [22]. Key cellular components of PVS involving perivascular fibroblasts (PVFs) and perivascular macrophages (PVMs)have been implicated in the pathogenesis of ALS [22,23]. However, the underlying mechanisms remain unknown. It is essential to investigate the relationship between EPVS and ALS in order to further understand the mechanisms and explore potential markers.

Genomic approaches are powerful tools for investigating causal relationships and revealing underlying potential mechanisms. Although a recent Mendelian randomization (MR) analysis conducted by Wang et al. revealed no causal link from EPVS to ALS, this was a limited observation as it lacked complementary genetic analyses and did not use the most recent ALS GWAS [24]. Therefore, to clarify this relationship, we performed a systematic genetic investigation utilizing the largest and most recent GWAS dataset.

## 2. Materials and Methods

### 2.1. Study Design

This study aimed to investigate the shared genetic link between EPVS and ALS based on the GWAS summary data. First, pairwise genetic correlation was evaluated by the linkage disequilibrium score regression (LDSC). Second, the cross-trait GWAS meta-analyses, including multi-trait analysis of GWAS (MTAG) and Cross-Phenotype Association (CPASSOC) analysis, were employed to identify potential shared risk single nucleotide polymorphisms (SNPs). Then, genetic colocalization analysis using Bayesian algorithms was conducted to determine whether two traits share the same genetic information in a given area. Finally, two-sample bidirectional MR analysis was used to investigate potential causal relationships. The study was conducted in accordance with the Declaration of Helsinki.

### 2.2. Data Source

We used summary statistics retrieved from publicly available GWAS of individuals with European ancestry. For ALS, we used GWAS data from GCST90027164, which included 27,205 patients with ALS and 110,881 control participants [9]. All patients were diagnosed with definite, probable or possible ALS according to the revised EI Escorial criteria by neurologists at specialized clinics. Control participants were drawn from general population cohorts without ALS diagnoses. These case and control samples provided genome-wide association estimates for ALS risk across the population.

For EPVS, we used summary statistics from a recent GWAS meta-analysis that involved 40,095 participants from population-based cohorts [25]. The EPVS burden was assessed on MRI in multiple brain regions, including in the hippocampus (EPVS-HIP), basal ganglia (EPVS-BG), and white matter (EPVS-WM). EPVS-HIP was observed in 8950 out of 38,871 participants. EPVS-BG was observed in 9189 out of 38,903 participants. EPVS-WM was observed in 9317 out of 38,598 participants. Individuals were categorized by the extent of EPVS counts, with higher versus lower burden used as the outcome for analyses. All analyses in this study were performed using these summary statistics.

### 2.3. Data Analysis

To explore the genetic relationship between EPVS and ALS, we performed several statistical methods using GWAS summary statistics.

### 2.4. Genetic Correlation Analysis

LDSC analysis can assess genetic correlations between GWAS data [26,27]. LDSC quantifies the genetic overlap between two traits at an aggregate level, through estimates of heritability (h^2^) and genetic correlation (r_g_). Furthermore, it is not affected by environmental confounders. We performed LDSC to investigate the genetic correlation between EPVS and ALS. LDSC analysis was conducted using summary statistics from largely independent cohorts. SNPs with minor allele frequencies (MAFs) ≤ 0.01 were excluded. Linkage disequilibrium (LD) scores were precomputed based on the European-ancestry samples of the 1000 Genomes Project. A Bonferroni-corrected p threshold of 0.05/3 = 1.67 × 10^−2^ was considered to indicate statistical significance.

### 2.5. Shared Risk Loci Analysis

In order to identify genetic variants associated with both EPVS and ALS, we performed cross-trait meta-analyses including MTAG and CPASSOC. MTAG can increase the power to detect genetic associations shared across traits while preserving trait-specific effect estimates [28]. The analysis was restricted to SNPs with MAFs > 0.01. As the EPVS and ALS GWAS were largely derived from independent cohorts, MTAG was applied under the assumption of minimal sample overlap. CPASSOC is a statistical method used to identify pleiotropic genetic variants which can simultaneously affect different phenotypes and regions of the genome that may play a role in related biological processes [29]. CPASSOC provides two statistical approaches including Shom (employs a fixed-effects meta-analysis, which is appropriate for detecting homogeneous genetic effect sizes) and SHet (extends SHom by increasing sensitivity to heterogeneous effects). Due to its enhanced statistical stability and power, we used SHet to integrate the summary data of EPVS and ALS. PLINK1.9 was used to perform clumping procedures across a range of 500 kb and r^2^ > 0.2 [30]. SNPs exhibiting *p* < 0.05 in the single-trait analysis and *p* < 5 × 10^−8^ in the CPASSOC analysis were considered to be significant.

### 2.6. Genetic Colocalization Analysis

The Coloc package (version 5.2.3) was used to perform genetic colocalization analysis. The genetic colocalization analysis can determine whether a single genetic variant could affect both EPVS and ALS in the same region of the genome [31]. Within the genetic colocalization analysis, posterior probabilities (PPs) were calculated for the following five hypotheses: (1) PPs of hypothesis 0 (PPH0), association with neither trait 1 nor trait 2; (2) PPs of hypothesis 1 (PPH1), association with trait 1 but not with trait 2; (3) PPs of hypothesis 2 (PPH2), association with trait 2 but not with trait 1; (4) PPs of hypothesis 3 (PPH3), association with both trait 1 and trait 2, but via independent single nucleotide variants (SNVs); and (5) PPs of hypothesis 4 (PPH4), association with trait 1 and trait 2 via shared SNVs. In line with previous studies, we considered a PPH4 of 0.7 or more as indicating strong evidence for genetic colocalization [32].

### 2.7. Bidirectional Mendelian Randomization Analysis

MR analysis uses genetic variants as instruments to assess whether one trait causally influence another at the genetic level. Here, we performed a bidirectional MR analysis to assess the potential causal relationship between EPVS and ALS. Since it remains unclear whether EPVS influence ALS risk or whether ALS influences EPVS burden, this bidirectional approach allowed us to separately assess the effect of EPVS on ALS risk and the effect of ALS on EPVS burden. In MR, genetic variants that are closely associated with the exposure and independent of the outcome were selected as instrumental variables (IVs). For independent IVs, we performed clumping (r^2^ < 0.1, distance = 1000 kb) of SNPs that exhibited genome-wide significance (*p* < 5 × 10^−8^). For lead SNPs that were not available in the outcome dataset, we used the SNiPA platform (https://snipa.helmholtz-munich.de/index.php?task=proxy_search; accessed on 26 November 2025).to search for proxy SNPs in high linkage disequilibrium (r^2^ > 0.8). Principal MR methods including inverse variance weighting (IVW), weighted median, simple median and MR-Egger analyses were conducted. We generated forest plots and scatter plots to visualize overall effects and individual SNP effects. Sensitivity analyses including the MR-Egger intercept test, Cochran’s Q statistic and the MR pleiotropy residual sum and outlier test (MR-PRESSO), were conducted to assess horizontal pleiotropy and heterogeneity [33]. MR-PRESSO was performed with 10,000 permutations and involves three tests: (1) a global test for assessing horizontal pleiotropy; (2) an outlier test to identify individual SNPs that disproportionately contribute to heterogeneity; and (3) a distortion test to evaluate whether the removal of outliers affects the causal estimate. If an outlier SNP was identified (*p* < 0.05), causal estimates were reported before and after the removal of outliers. We also performed the “leave-one-out” method to investigate whether the causality estimates were attributable to specific SNPs. “Two-Sample MR” and “MR-PRESSO” packages in R software (version 4.3.0) were used in this study.

### 2.8. Institutional Review Board Statement/Informed Consent Statement

This study was based on publicly available GWAS data and did not directly involve human participants. Ethical approval and written informed consent from all participants were presented from the original studies and the data source websites (GWAS-PVS: https://doi.org/10.1038/s41591-023-02268-w; GWAS-ALS: https://doi.org/10.1038/s41588-021-00973-1). Therefore, no additional informed consent was required.

## 3. Results

**Figure 1:**
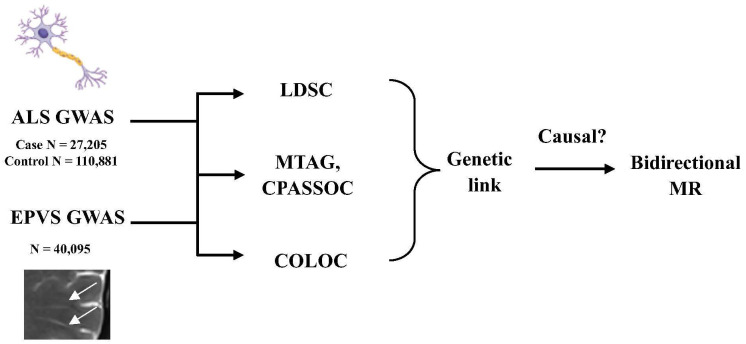
Flowchart of this study. Abbreviation: ALS, amyotrophic lateral sclerosis; GWAS, genome-wide association study; EPVS, enlarged perivascular spaces; LDSC, linkage disequilibrium score regression; MTAG, multi-trait Analysis of GWAS; CPASSOC, Cross-Phenotype Association; COLOC, colocation; MR, mendelian randomization.

The study flowchart is shown in Figure 1. First, we performed LDSC analysis to investigate the genetic correlation between EPVS across distinct regions and ALS. The results including the SNP h^2^ of EPVS and ALS are shown in Table 1. A significant negative genetic correlation (r_g_ = −0.24, *p* < 0.01) was observed between EPVS-WM and ALS after correction for multiple testing. There were no significant correlations detected between EPVS-HIP (r_g_ = −0.03, *p* = 0.79), EPVS-BG (r_g_ = 0.01, *p* = 0.91) and ALS.

**Figure 2:**
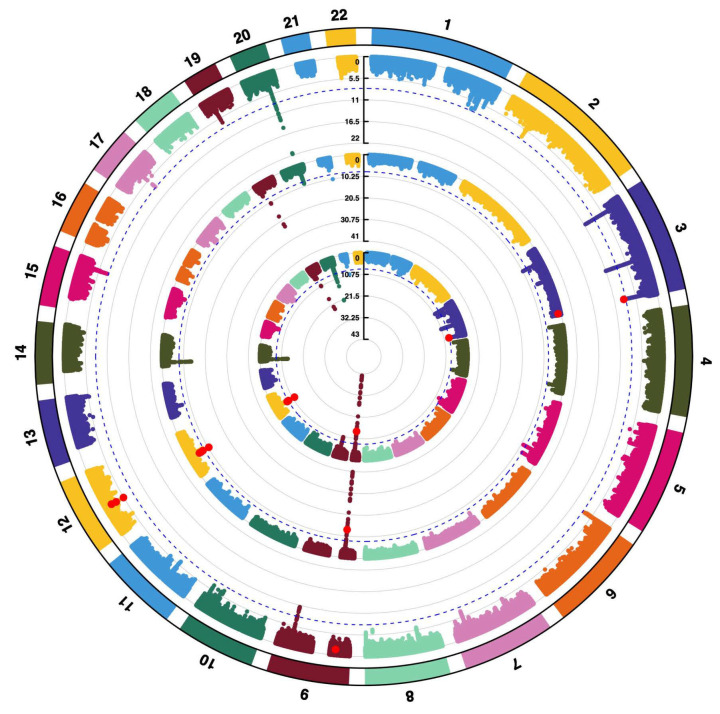
Circular Manhattan plot showing potential shared genetic association signals between EPVS-WM and ALS. Each point represents a SNP. SNPs are arranged by genomic position and statistical significance is indicated by −log_10_(P) values. The innermost ring shows the genome-wide *p*-values from the cross-trait association analysis between ALS and EPVS-WM, with genome-wide significant SNPs (*p* < 5 × 10^−8^) marked in red. The middle ring shows the *p*-values adjusted for the genetic effects of ALS. SNPs (*p* < 0.05) are marked in red. The outermost ring shows the *p*-values adjusted for the genetic effects of EPVS. SNPs with *p* < 0.05 are marked in red. Abbreviation: EPVS, enlarged perivascular spaces; WM, white matter; ALS, amyotrophic lateral sclerosis; SNP, single nucleotide polymorphism.

Second, given the significant genetic correlation detected between EPVS-WM and ALS, we performed a cross-trait meta-analysis to investigate the associated loci. We performed MTAG analysis to improve the statistical power. Then, we performed a CPASSOC analysis to identify specific loci driving the relationship. Circular Manhattan plot was used to visualize the results (Figure 2). Significant loci (*p* < 0.05 for single trait analysis; *p* < 5 × 10^−8^ for meta-analysis) are shown in red. Seven loci were observed to be significant for both EPVS and ALS. Four genome-wide significant loci were identified to be potentially shared between EPVS and ALS (Table 2). Two of these loci were mapped to protein-coding genes. In these 2 loci, a stronger signal was observed on chromosome 12 at the *KIF5A* region (lead SNP rs113247976, *p* = 2.106 × 10^−12^). The other signal was detected on chromosome 12 in the *SDR9C7* region (lead SNP rs118082508, *p* = 1.767 × 10^−8^).

Third, we performed a genetic colocalization analysis between EPVS-WM and ALS (Table 3). The results did not support genetic colocalization between them. The results of PPH4 were very low, thus indicating an absence of evidence for either shared or distinct causal variants.

**Figure 3:**
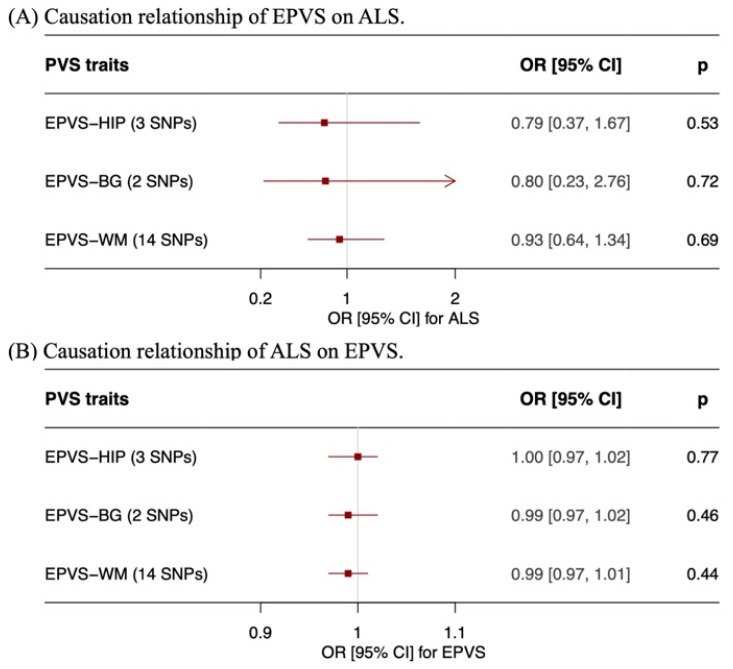
Forest plots showing the results of bidirectional Mendelian randomization analyses investigating the causal relationship between EPVS and ALS. (**A**) forward Mendelian randomization evaluating the effect of EPVS on ALS risk, (**B**) reverse Mendelian randomization evaluating the effect of ALS on EPVS. Points represent OR, and horizontal lines indicate 95% CI. Abbreviation: EPVS, enlarged perivascular spaces; ALS, amyotrophic lateral sclerosis; HIP, hippocampus; BG, basal ganglia; WM, white matter; OR, Odds Ratio; CI, Confidence Interval.

Finally, a bidirectional MR analysis was performed to investigate the causal relationship between EPVS and ALS (Table 4). In the forward MR, we employed EPVS as exposures and investigated its causal effect on ALS. We extracted 3 SNPs associated with EPVS-HIP, 2 SNPs associated with EPVS-BG, and 14 SNPs associated with EPVS-WM. The numbers of SNPs of EPVS-HIP and EPVS-BG were so insufficient that some complex MR analyses (such as the MR-Egger test) could not be performed. Only IVW was performed to assess the effects of EPVS-HIP and EPVS-BG on ALS. The results revealed that there were not significant causal effects of EPVS-HIP, EPVS-BG or EPVS-WM on ALS. The forest and scatter plots are displayed in Figure 3 and Figure S1.

The results of the sensitivity analyses, including pleiotropy and heterogeneity analyses, are shown in Table 5. The MR-PRESSO Global Test suggested potential horizontal pleiotropy of EPVS-WM on ALS (RSSobs = 27.14, *p* = 0.04), and 1 outlier (rs6011998) was identified. The removal of this SNP did not substantially alter the causal estimate (distortion coefficient = −565.06, *p* = 0.06), and the outlier-corrected effect remained nonsignificant (β = 0.02, SE = 0.16, *p* = 0.92). Consistent with this, the MR-Egger intercept was not statistically significant (intercept = −0.02, *p* = 0.21), suggesting no evidence of directional horizontal pleiotropy. MR-PRESSO and MR-Egger analyses could not be performed to determine the effect of EPVS-HIP and EPVS-BG on ALS because of the insufficient IVs.

Leave-one-out analysis revealed that the exclusion of the detected outlier (rs6011998) or any other single SNP did not change the causal estimate of EPVS-WM on ALS (Figure S2). Cochran’s Q test indicated potential heterogeneity of the effects of EPVS-WM on ALS (Q = 22.77, *p* = 0.03), thus suggesting variability in the SNP-specific estimates. There was no heterogeneity detected in the effects of EPVS-HIP (Q = 0.11, *p* = 0.74) or EPVS-BG (Q = 1.96, *p* = 0.16) on ALS. Taken together, although potential heterogeneity was present, the overall pattern observed across the MR-PRESSO, MR-Egger, and leave-one-out analyses did not support a robust causal effect of EPVS-WM for ALS, and the results appeared to be stable after outlier removal. The IVW results showed no evidence of causal effects between EPVS-HIP, EPVS-BG and ALS.

Subsequently, reverse MR analyses were performed by employing ALS as an exposure and EPVS burden in distinct regions as outcomes (Table 4 and Table 5). Fifteen SNPs were extracted to be associated with ALS. The IVW results, as well as the results of the simple median, weighted median and MR Egger tests, were observed to be in agreement. The results showed that there was no evidence of causal effects of ALS on EPVS-HIP, EPVS-BG or EPVS-WM. Cochran’s Q statistic indicated no observable heterogeneity. The MR-PRESSO global test and MR-Egger intercept presented no evidence of horizontal pleiotropy of ALS on EPVS-HIP, EPVS-BG or EPVS-WM. Leave-one-out analysis suggested no evidence of correlation driven by a single SNP (Figure S3).

## 4. Discussion

In the present study, we used the largest and most recent GWAS dataset for ALS (*n* = 138,086) and applied multiple genetic approaches involving LDSC, MTAG, CPASSOC, loci-level genetic colocalization analysis and bidirectional MR analysis to investigate the shared genetic link between EPVS burden and ALS. We identified a significant negative genetic correlation between EPVS-WM burden and ALS. Loci including rs113247976 in *KIF5A* and rs118082508 in *SDR9C7* were identified as potential pleiotropic loci shared between EPVS-WM burden and ALS. However, neither the bidirectional MR analysis nor the genetic colocalization analysis revealed any significant causal relationship between EPVS and ALS. Therefore, our findings suggest that EPVS-WM burden and ALS may share some genetic architecture, although no evidence for a causal relationship was observed.

EPVS were initially regarded as imaging markers of cerebral small vessel disease and associated with reduced blood flow and oxidative stress [34,35]. Recent evidence suggests that EPVS are involved not only in these vascular pathophysiological processes but also in interstitial fluid exchange and waste clearance, as a critical component of the glymphatic drainage system [11,13]. Evidence regarding the distinct distribution of EPVS and their relationship with ALS remains limited and inconsistent [36,37]. Previous studies have suggested that EPVS abnormalities in ALS may specifically involve the white matter. Lee et al. reported a greater EPVS-WM burden in ALS patients compared with controls [36]. They further demonstrated that cervical lymphatic ligation in *SOD1*/G93A ALS mice resulted in an increase in PVS width and misfolded SOD1 accumulation, thereby linking EPVS to impaired clearance of pathological proteins. In contrast, other studies reported no differences in EPVS-WM burden between ALS patients and controls [37]. In addition, Wang et al. have recently conducted an MR analysis study to investigate the causal estimates of EPVS on neurodegenerative diseases. They reported no evidence of causal relationships between EPVS burden and ALS risk [24]. However, they reported that a higher EPVS-WM burden was associated with a lower risk of Alzheimer’s disease (AD).

In this study, EPVS were assessed in the basal ganglia, white matter and hippocampus. These brain regions are the most frequently assessed locations in neurodegenerative disease research, and they are currently the only areas with available GWAS data [25,38,39,40]. Future studies including EPVS data in other regions will be important to extend these findings.

When integrating these findings with our results, several factors may explain the apparent prominence of EPVS in the white matter. From an anatomical perspective, the white matter consists predominantly of a high density of axonal tracts, thus is vulnerable to axonal transport dysfunction and protein aggregation, which are the key mechanisms of ALS [41]. Furthermore, the densely arranged architecture results in a relatively uniform extracellular environment compared to the more heterogeneous gray matter. In addition, the white matter is vascularized by penetrating arteries with limited circulation [42]. The interstitial fluid drainage of the white matter may be less efficient than that in cortical regions, thus EPVS-WM are thought to be more closely related to impaired glymphatic clearance [43,44]. In contrast, EPVS-BG are more strongly associated with hypertensive arteriopathy and arterial pulsatility dysfunction, while the mechanisms underlying EPVS-HIP remain poorly understood [43,45]. Taken together, the unique anatomical features of white matter likely lead to its distinctive pathological susceptibility to ALS and to the visibility of EPVS. Although EPVS are generally considered connected to the subarachnoid space, evidence for functional differences in communication between EPVS-WM and those in other regions remains limited. Genetic studies further support these regional differences. EPVS burden exhibits region-specific genetic architecture, with EPVS-WM demonstrating the highest heritability, and EPVS-BG and EPVS-HIP exhibiting relatively low genetic correlation [25].

The observed genetic link between a higher EPVS-WM burden and a lower risk of ALS should be interpreted with caution. First, current GWAS on EPVS mainly focused on EPVS count rather than their size or volume. These features may also be relevant to EPVS function [13,34]. Second, EPVS features appear to be dynamic and may follow a non-linear pattern [46]. However, genetic analyses only reflect lifelong susceptibility rather than stage-specific changes. Third, the finding may be associated with the compensatory glymphatic responses. Increase in EPVS count may accelerate glymphatic clearance [24]. Consequently, the genetic and imaging findings may not always align directly. Future studies including automated measures of the number, size and volume of EPVS are needed.

SNPs including rs113247976 in *KIF5A* and rs118082508 in *SDR9C7* were identified as potential pleiotropic loci associated with both EPVS and ALS. However, genetic colocalization analyses did not support a shared causal variant at these loci. These findings suggest statistical pleiotropy rather than a shared causal mechanism. *KIF5A* encodes a kinesin motor protein involved in axonal transport [47,48], and mutations in *KIF5A* have been reported to be associated with ALS [49,50]. The lack of genetic colocalization suggests that distinct variants within the *KIF5A* locus may independently influence EPVS and ALS risk. Although no shared causal variant was identified, previous studies have implicated disrupted protein homeostasis in ALS, while EPVS are generally thought to reflect impaired clearance of interstitial waste [11,51]. These observations describe related but biologically distinct processes. This hypothesis remains speculative and is not directly supported by our genetic analyses, but it provides a potential clue for future studies.

Notably, the association between *SDR9C7* and ALS has not been previously reported. *SDR9C7* encodes a member of the short-chain dehydrogenases/reductase superfamily [52]. Given the established role of oxidative stress in ALS, *SDR9C7* may warrant further investigation [51]. However, without evidence of shared causal variant, the observed associations should be interpreted cautiously. Further genetic and functional studies are needed to clarify their roles in EPVS and ALS.

Some limitations in this study should be noted. First, we included only individuals of European ancestry to avoid genetic ancestry bias, which may restrict the generalizability of our findings. Second, the limited number of available SNPs for EPVS-BG and EPVS-HIP reduced the robustness of the MR analyses, leading to lower statistical power and an increased risk of false-negative findings. In addition, due to the small number of IVs, several sensitivity analyses could not be reliably performed. Therefore, null results for these regions should be interpreted cautiously. Although more relaxed significance thresholds could increase the number of IVs, this approach may increase the risk of weak instrument bias and horizontal pleiotropy. Third, although the EPVS and ALS GWAS were largely based on independent cohorts, limited sample overlap cannot be completely excluded. Finally, MR analysis is designed to assess linear relationships, therefore non-linear causal relationships could not be detected via MR. Moreover, while MR can suggest potential causal relationships at the genetic level, it cannot determine the sequence of development. Follow-up longitudinal studies are necessary to address this limitation. In addition, although the genetic correlation between EPVS-WM and ALS remained significant after correction for multiple testing, false-positive findings cannot be completely ruled out. Therefore, these findings should be interpreted with caution, and future studies with larger sample sizes or stronger genetic instruments are needed to validate these conclusions.

## 5. Conclusions

In this study, we conducted a comprehensive analysis of the genetic relationship between EPVS and ALS. We observed a negative genetic correlation between EPVS-WM and ALS, although evidence for a causal effect was lacking. *KIF5A* and *SDR9C7* were identified as potential loci involved in this shared genetic architecture. These findings enhance our understanding of the genetic mechanisms underlying both EPVS and ALS. Future research is required to further clarify these findings.

## Figures and Tables

**Table 1:** The genetic correlation between EPVS across distinct regions and ALS.

		Heritability	Genetic Correlation with ALS		
Traits	Sample Size	h^2^	r_g_	SE	*p*
EPVS-HIP	38,871	0.039	−0.03	0.10	0.79
EPVS-BG	38,903	0.038	0.01	0.12	0.91
EPVS-WM	38,598	0.038	−0.24	0.08	<0.01 *
ALS	138,086	0.038			

Abbreviation: EPVS, enlarged perivascular spaces; ALS, amyotrophic lateral sclerosis; HIP, hippocampus; BG, basal ganglia; WM, white matter; r_g_, genetic correlation estimate; SE, Standard Error. * *p* < 0.05.

**Table 2:** Cross-trait meta-analysis results between EPVS-WM and ALS.

SNP	hg19_ Coordinates	a1	A1 freq	p_shet	Consequence	hgnc	Gene Function
rs10967993	chr9:27582311	T	0.4887	5.503 × 10^−15^	NA	NA	NA
rs113247976	chr12:57975700	T	0.0145	2.106 × 10^−12^	missense	KIF5A	Protein coding gene
rs9683037	chr3:193529913	A	0.7161	5.366 × 10^−9^	NA	NA	NA
rs118082508	chr12:57318819	T	0.0155	1.767 × 10^−8^	intron	SDR9C7	Protein coding gene

Abbreviation: EPVS, enlarged perivascular spaces; ALS, amyotrophic lateral sclerosis; WM, white matter.

**Table 3:** Genetic colocalization analysis results between EPVS and ALS.

SNP	PPH0	PPH1	PPH2	PPH3	PPH4
rs10967993	8.31 × 10^−37^	2.60 × 10^−38^	9.66 × 10^−1^	3.03 × 10^−2^	3.36 × 10^−3^
rs113247976	1.78 × 10^−5^	2.65 × 10^−7^	9.19 × 10^−1^	1.36 × 10^−2^	6.77 × 10^−2^
rs9683037	8.78 × 10^−1^	1.26 × 10^−2^	1.07 × 10^−1^	1.54 × 10^−3^	8.77 × 10^−4^
rs118082508	1.78 × 10^−5^	2.65 × 10^−7^	9.19 × 10^−1^	1.36 × 10^−2^	6.77 × 10^−2^

Abbreviation: EPVS, enlarged perivascular spaces; ALS, amyotrophic lateral sclerosis; SNP, single nucleotide polymorphism; PPH0, posterior probability of hypothesis 0; PPH1, posterior probability of hypothesis 1; PPH2, posterior probability of hypothesis 2; PPH3, posterior probability of hypothesis 3; PPH4, posterior probability of hypothesis 4.

**Table 4:** Bidirectional Mendelian randomization (MR) analysis between EPVS and ALS. (A) The causal effect of EPVS (exposure) on the risk of ALS (outcome). (B) The causal effect of ALS (exposure) on the risk of EPVS (outcome).

**(A)**									
**Outcome**	**Exposures**	**Inverse-Variance Weighted**		**Simple** **Median**		**Weighted** **Median**		**MR-Egger**	
		**OR** **[95%CI]**	***p***	**OR** **[95%CI]**	***p***	**OR** **[95%CI]**	***p***	**OR** **[95%CI]**	***p***
	EPVS-HIP ^#^	0.79 [0.37, 1.67]	0.53	/	/	/	/	/	/
ALS	EPVS-BG ^#^	0.80 [0.23, 2.76]	0.72	/	/	/	/	/	/
	EPVS-WM	0.93 [0.64,1.34]	0.69	0.89 [0.57, 1.40]	0.62	0.98 [0.65, 1.46]	0.90	1.65 [0.66,4.13]	0.31
**(B)**									
**Outcomes**	**Exposure**	**Inverse-Variance Weighted**		**Simple** **Median**		**Weighted** **Median**		**MR-Egger**	
		**OR** **[95%CI]**	***p***	**OR** **[95%CI]**	***p***	**OR** **[95%CI]**	***p***	**OR** **[95%CI]**	***p***
EPVS-HIP		1.00 [0.97, 1.02]	0.77	0.99 [0.95, 1.03]	0.63	0.99 [0.96, 1.02]	0.54	1.00 [0.93, 1.07]	0.96
EPVS-BG	ALS	0.99 [0.97, 1.02]	0.46	1.00 [0.97, 1.04]	0.96	1.00 [0.97,1.03]	0.94	1.01 [0.96,1.08]	0.66
EPVS-WM		0.99 [0.97, 1.01]	0.44	0.99 [0.96, 1.02]	0.41	1.00 [0.96, 1.03]	0.84	1.00 [0.94, 1.06]	0.95

# No enough instrumental variables for Simple median, Weighted median and MR-Egger. MR estimates indicate potential causal relationships at the genetic level. Abbreviation: MR, mendelian randomization; EPVS, enlarged perivascular spaces; ALS, amyotrophic lateral sclerosis; HIP, hippocampus; BG, basal ganglia; WM, white matter; OR, Odds Ratio; CI, Confidence Interval.

**Table 5:** The estimations of heterogeneity and horizontal pleiotropy for MR results. (A) The causal effect of EPVS(exposure) on the risk of ALS (outcome). (B) The causal effect of ALS (exposure) on the risk of EPVS (outcome).

**(A)**						
**Outcome**	**Exposures**			**MR-Egger**		**MR-PRESSO**
		**Cochran Q**	***p***	**Egger Intercept**	***p***	***p*** **for Global Test**
	EPVS-HIP ^#^	0.11	0.74	/	/	/
ALS	EPVS-BG ^#^	1.96	0.16	/	/	/
	EPVS-WM	22.77	0.03 *	−0.02	0.21	0.04 *
**(B)**						
**Outcomes**	**Exposure**			**MR-Egger**		**MR-PRESSO**
		**Cochran Q**	***p***	**Egger Intercept**	***p***	***p*** **for Global Test**
EPVS-HIP		13.65	0.25	−0.0002	0.95	0.27
EPVS-BG	ALS	8.91	0.63	−0.002	0.43	0.64
EPVS-WM		7.70	0.74	−0.001	0.69	0.79

# No enough instrumental variables for MR-Egger and MR-PRESSO. MR estimates indicate potential causal relationships at the genetic level. Abbreviation: MR, mendelian randomization; EPVS, enlarged perivascular spaces; ALS, amyotrophic lateral sclerosis; HIP, hippocampus; BG, basal ganglia; WM, white matter. * *p* < 0.05.

## Data Availability

The data supporting the findings of this study are publicly available from data source websites (GWAS-PVS: https://doi.org/10.1038/s41591-023-02268-w; GWAS-ALS: https://doi.org/10.1038/s41588-021-00973-1). Codes used in the study are available from the corresponding authors by request.

## Supplementary Materials

The following supporting information can be downloaded at: https://www.mdpi.com/article/10.3390/brainsci16020144/s1, Figure S1: Scatter plots of the association between EPVS and ALS in the MR analysis; Figure S2: Leave-one-out sensitivity analysis assessing the robustness of MR results for the causal effect of white matter EPVS burden on ALS; Figure S3: Leave-one-out sensitivity analysis assessing the robustness of MR results for the causal effect of ALS on EPVS burden.

## Abbreviations

The following abbreviations are used in this manuscript:

ALS	Amyotrophic lateral sclerosis
EPVS	Enlarged perivascular space
EPVS-HIP	EPVSs in the hippocampus
EPVS-BG	EPVSs in the basal ganglia
EPVS-WM	EPVSs in the white matter
LDSC	Linkage disequilibrium score regression
MTAG	Multi-Trait Analysis of GWAS
CPASSOC	Cross-Phenotype Association
GWAS	Genome-wide association study
MRI	Magnetic resonance imaging
PVFs	Perivascular fibroblasts
PVMs	Perivascular macrophages
MR	Mendelian randomization
SNPs	Single Nucleotide Polymorphisms
LD	Linkage disequilibrium
MAF	Minor allele frequency
PPs	Posterior probabilities
IVs	Instrumental variables
SNVs	Single nucleotide variants
IVW	Inverse variance weighting
MR-PRESSO	MR pleiotropy residual sum and outlier test
