# Supplementary material for: Enlarged Perivascular Spaces (EPVS) and the Risk of Amyotrophic Lateral Sclerosis (ALS): Evidence for Overlapping Genetic Signals in White Matter Without Causal Links
Source: Brain Sci. 2026 Jan 28;16(2):144. doi: 10.3390/brainsci16020144 (PMC12938650; doi:10.3390/brainsci16020144)
Supplement: Supplementary file 1 [file brainsci-16-00144-s001.zip › brainsci-4079845-supplementary.pdf]

Supplementary materials

Investigating the shared genetic link of enlarged perivascular spaces and amyotrophic lateral sclerosis: A genome-wide cross trait analysis and Mendelian randomization study.

Xin Huang[1†], Kailin Xia[1,2,3†], Shan Ye[1,2,3], Qiong Yang[1,2,3#], Dongsheng Fan[1,2,3#]

1. Department of Neurology, Peking University Third Hospital, Beijing, China.
2. Key Laboratory for Neuroscience, National Health Commission/Ministry of Education, Peking University, Beijing, China.
3. Beijing Key Laboratory of Biomarker and Translational Research in Neurodegenerative Diseases, Beijing, China.

Figure S1: Scatter plots of the association between EPVS and ALS in the MR analysis.

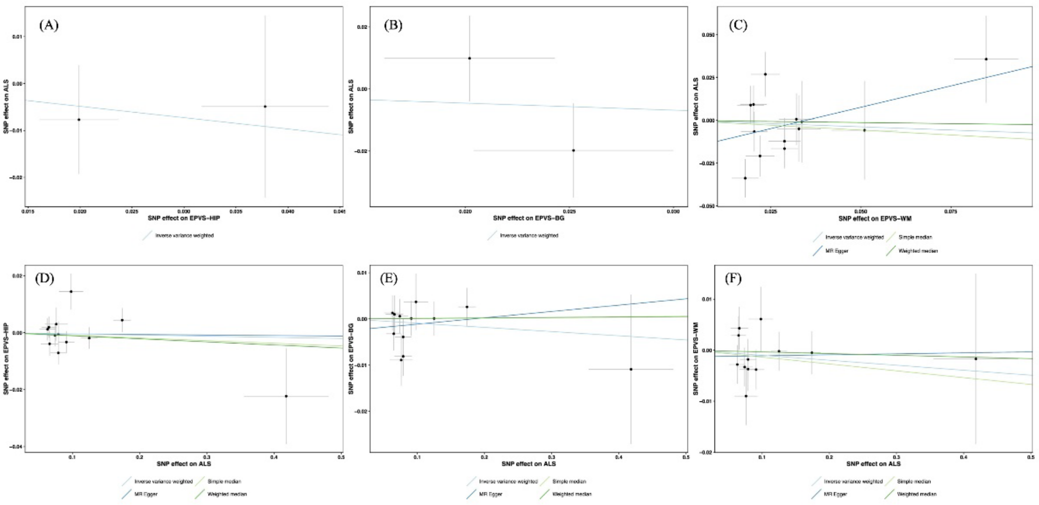

(A) effect of hippocampal EPVS on ALS, (B) effect of basal ganglia EPVS on ALS, (C) effect of white matter EPVS on ALS, (D) effect of ALS on hippocampal EPVS, (E) effect of ALS on basal ganglia EPVS, (F) effect of ALS on white matter EPVS.

Abbreviation: EPVS, enlarged perivascular spaces; ALS, amyotrophic lateral sclerosis； MR, mendelian randomization.

Figure S2: Leave-one-out sensitivity analysis assessing the robustness of MR results for the causal effect of white matter EPVS burden on ALS.

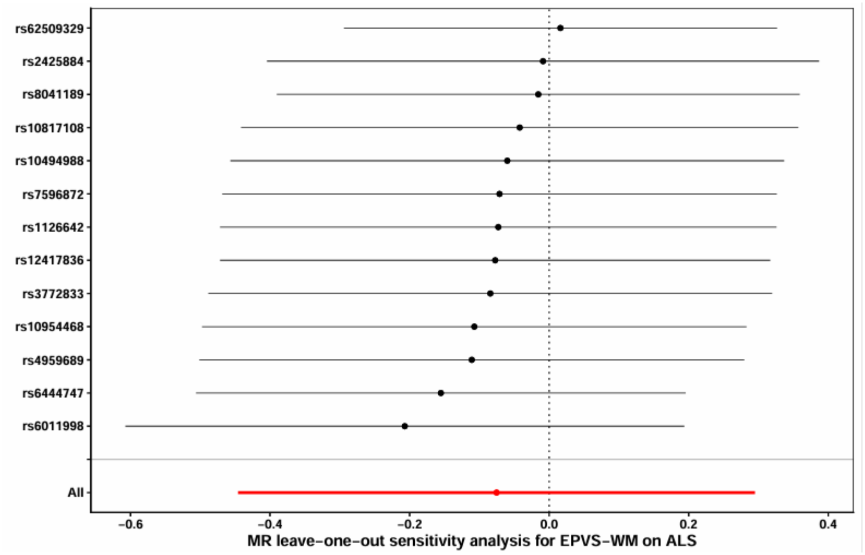

Abbreviation: MR, mendelian randomization; EPVS, enlarged perivascular spaces; ALS, amyotrophic lateral sclerosis.

Figure S3: Leave-one-out sensitivity analysis assessing the robustness of MR results for the causal effect of ALS on EPVS.

(A)

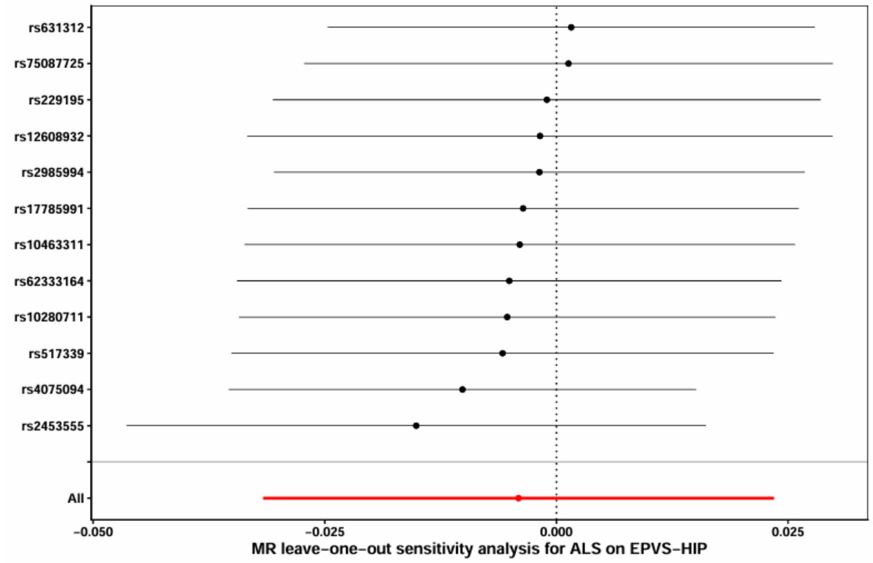

(B)

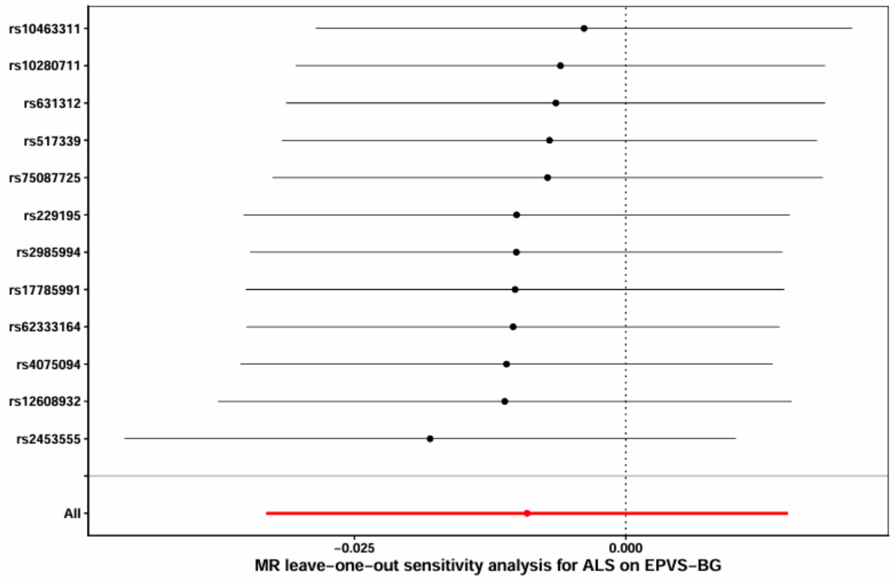

(C)

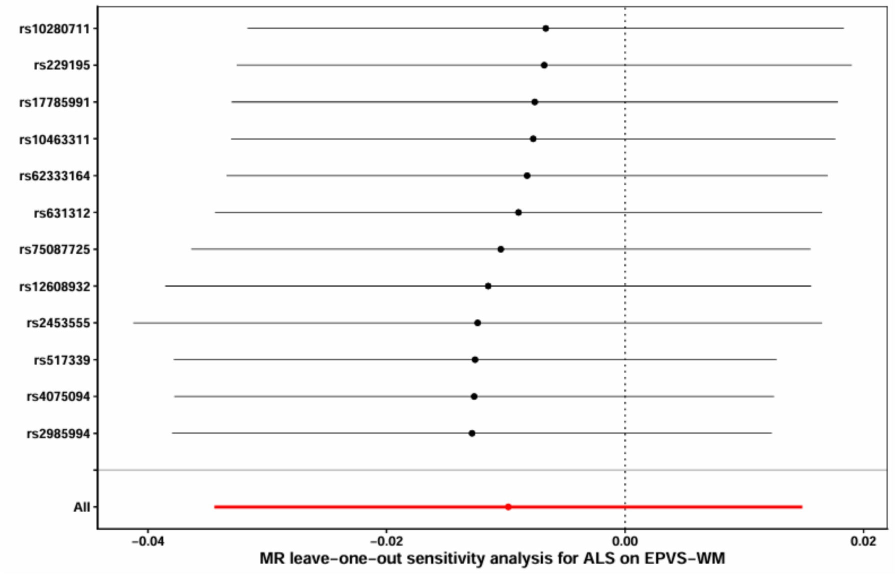

(A) hippocampal EPVS burden, (B) basal ganglia EPVS burden, and (C) white matter EPVS burden. Abbreviation: MR, mendelian randomization; EPVS, enlarged perivascular spaces; ALS, amyotrophic lateral sclerosis.
